# ZIC2 promotes cancer stem cell traits via up-regulating OCT4 expression in lung adenocarcinoma cells

**DOI:** 10.7150/jca.44367

**Published:** 2020-08-19

**Authors:** Wang Wei-Hua, Zhou Ning, Chen Qian, Jiang Dao-Wen

**Affiliations:** 1 Department of thoracic surgery, Minhang Hospital, Fudan University, Shanghai 201100, China.; 2 Department of general surgery, Minhang Hospital, Fudan University, Shanghai 201100, China.

**Keywords:** lung adenocarcinoma, cancer stem cells, ZIC2, OCT4 activation

## Abstract

**Background:** Accumulating evidence has revealed the importance of cancer stem cells (CSCs) in self-renewal and chemoresistance. Previous studies reported high expression of ZIC2 was closely associated with tumorigenesis and CSC traits. However, the role of ZIC2 as a crucial factor for regulating CSC properties in lung adenocarcinoma (LAC) remains elusive.

**Methods:** RT-PCR and WB assay were employed to assess ZIC2 expression in 20 LAC tumor tissues and the matched non-cancerous tissues. The role of ZIC2 in LAC CSC were analyzed by evaluation of CSC-related markers expression and spheroid formation *in vitro*. Cisplatin and paclitaxel resistance capacities were evaluated by CCK8 assay, colony formation assay, and flow cytometry analysis. Subcutaneous NOD/SCID mice models were generated to assess *in vivo* CSC features.

**Results:** High expression of ZIC2 was found in LAC tumor tissues and indicated a poor overall survival in LAC patients. ZIC2 upregulated an array of CSCs-related genes, including EpCAM, OCT4, SOX2, NANOG, C-Myc and Bmi-1. Knockdown of ZIC2 inhibited sphere-forming capacity and decreased cisplatin and paclitaxel resistance. However, overexpression of ZIC2 achieved opposite effects. Mechanically, ZIC2 acts upstream of OCT4 to promote its expression, resulting in enhancement of CSC traits in LAC.

**Conclusion:** Our results demonstrated that ZIC2 was crucial for promoting CSC traits in LAC cells, and served as a potential biomarker for predicting prognosis. The ZIC2-OCT4 network will facilitate the evaluation of the potential therapeutic efficacy of chemotherapy and predict patient sensitivity to treatment.

## Introduction

Lung cancer is the major cause of cancer-related deaths worldwide 1-3. Lung adenocarcinoma (LAC) is listed as the most common subtypes of lung cancer, accounting for almost 40% 4-6. Distant metastasis and chemoresistance are primarily attributed to short survival for LAC patients 7. The cancer stem cells (CSCs) are defined as a rare subset of cancer cells and possesses ability to differentiate and self-renew 8. They are also responsible for tumor growth, cancerous recurrence, metastasis and chemoresistance 9. Therefore, understanding of the roles and mechanisms critical to lung CSC generation and expansion would improve the overall survival rates in LAC patients.

Transcription factor-mediated expression regulation is one of the critical regulatory mechanisms contributing to CSCs traits 10. OCT4, NANOG and SOX2 have been identified as the core pluripotent TFs in embryonic stem cells, which play important roles in establishing and maintaining the pluripotency of stem cells 11-13. OCT4, a member of the POU family, exerts a fundamental role in cancer stem cell self-renewal and multiple differentiation potential 14. Previous studies revealed that OCT4 was preferentially expressed in LAC stem cells and showed closely correlation with the generation and expansion of LAC CSCs 15-17. Additionally, OCT4 is critical to induce drug resistance in LAC patients 17. However, why OCT4 exhibited high expression in LAC stem cells remained unclear. Thus, it is essential to explore the regulatory mechanisms of OCT4 expression, which would contribute to understand the tumor genesis and drug resistance in LAC and obtain more potential therapeutic benefits in clinic.

The zinc finger family member 2 (ZIC2) functions as a transcriptional regulator and has been reported to be highly expressed in solid tumors 18. Importantly, high expression of ZIC2 is closely associated with tumorigenesis and self-renewal of cancer cells 19. Accumulating evidence indicated ZIC2 may be involved in lung cancer progression 20, 21. Interestingly, ZIC2-dependent OCT4 expression was involved in regulating CSC traits in liver cancer 19. However, the role of ZIC2 in LAC was still unknown. Here, we found that ZIC2 was upregulated in LAC tissues and indicates significantly poorer prognosis. Additionally, ZIC2 greatly enhanced sphere-forming, proliferation and chemoresistance capacities of LAC cells. Importantly, we revealed that ZIC2 was a key upstream regulatory gene of OCT4, which sustains the stemness of LAC CSCs.

## Materials and Methods

### Human tissue samples

Paired peri-tumor and tumor tissue specimens were obtained from 20 LAC patients in the Department of thoracic surgery, Minhang Hospital, Fudan University, for subsequent experiments. A pathological diagnosis was made according to the histology of tumor specimens or biopsy and was examined by experienced pathologists. All participants involved in this study provided informed consent before the commencement of the study.

### Cell lines

Normal lung cells HBEC and human LAC cell lines A549, NCI-H1975, PC9, NCI-H1650, NCI-H23, NCI-H1299 were purchased from Cell Bank of Type Culture Collection of Chinese Academy of Sciences (Shanghai, China). Cells were cultured in Dulbecco's Modified Eagle Medium (DMEM) (Gibco, USA) containing 10% fetal bovine serum (FBS) (Gibco, USA) and 100 units/ml of penicillin and 100 *µ*g/ml of streptomycin (Gibco, USA) in a humidified 37°C incubator with 5% CO_2_. According to the expression of ZIC2 in liver cell lines, we chose the ZIC2-high expressing LAC cell line NCI-H1299 to perform the loss-of-function experiments. The ZIC2 low-expressing LAC cell line NCI-H1975 was used to perform the gain-of-function experiments.

### Establishment of stable cell lines

Stable cell lines were established as previously described 18. Two shRNA sequences specifically against ZIC2 (shZIC2#1, shZIC2#2) and control-shRNA were used. Two distinct shRNAHuSH29-mershRNA constructs against ZIC2 in pGFP-V-RS vec-tor were purchased from Merdobio Technologies (Shanghai, China). The production of lentiviral particles and subsequent infection of NCI-H1299 and NCI-H1975 cells were performed. Positive cells were selected with puromycin (Clontech, Mountain View, CA, USA) for 10 days.

### RT-PCR

mRNA was extracted according to standard protocols provided by TRIzol (Invitrogen, USA). The total mRNA was reversed to cDNA followed by the PrimerScript^TM^ RT reagent Kit with gDNA Eraser (Perfect Real Time) (TaKaRa, China). Quantitative real-time PCR was performed with EvaGreen 2×qPCR MasterMix (Invitrogen, USA) in a CFX96^TM^ Real-Time PCR System (BioRad, USA). RT-qPCR was performed in triplicate. The relative levels of mRNA were normalized to those of *GAPDH*. The primer sequences used are listed in Supplemental Table 1.

### Western blot

Western blot was performed as previously described. In details, cultured cells were lysed in RIPA lysis buffer (Beyotime, China) supplemented with cocktail protease inhibitor (Roche, Switzerland). Proteins were separated by SDS-PAGE and were transferred onto polyvinylidene difluoride membranes (Millipore, USA). The membranes were blocked in 5% nonfat milk solution for 1 h at 37°C and were incubated with primary antibodies at 4°C overnight, followed by incubation with HRP-conjugated secondary antibodies (Beyotime, China) for 1 h. The signals were visualized using the BeyoECL Plus kit (Beyotime, China). The antibodies used can be found in Supplemental Table 2. GAPDH was used as an internal control.

### Cell viability assay

Cells (5 × 10^3^) were seeded in 96-well plates for 12 h, followed by treatment with cisplatin (Sigma, USA) at various concentrations (1, 5, 10, 20, 50, 100, 500, 1000 µM) and paclitaxel at various concentrations (0.001, 0.01, 0.1, 1, 10, 100 µm). After incubation for 48 h, cell viability was measured by both the 3-(4,5-dimethylthiazolyl-2]-2,5-diphenyltetrazolium bromide (MTT) (Sigma, St Louis, MO, USA) assay and Cell Count Kit-8 (CCK-8) (KeyGEN BioTECH, China) assay according to the manufacturer's instructions. The percentage of viable cells (%) = (absorbance of treated sample/absorbance of untreated sample) × 100%.

### Colony forming assay

Cells (400 cells/well) were seeded in 6-well plates. After 2 weeks of culture, colonies were fixed by 4% paraformaldehyde and were stained with 0.5% crystal violet. The number of colonies (≥100 µm in diameter) was counted with a microscope.

### Sphere forming assay

NCI-H1299 cells and NCI-H1975 cells were seeded in ultra-low attachment 6-well plates (Corning, USA) and cultured in DMEM/F12 supplemented with B27, 20 ng/ml EGF, 20 ng/ml bFGF, and 10 ng/ml HGF. Cells were incubated in a CO2 incubator 2 weeks later; spheres were counted under microscope (Olympus, Japan).

### Flow cytometry

Cells were treated with doxorubicin (0.5 µg/ml) for 48 h. Next, cells were harvested and stained in binding buffer, Annexin V-APC and 7-AAD as provided by the Annexin V-APC/7-AAD Apoptosis Detection Kit (KeyGEN BioTECH, China). Analysis was determined using a flow cytometer.

### Statistical analysis

All experiments were conducted at least in triplicate. All the data were presented as the means ± standard error of the mean (SEM). Statistical analyses were performed using Student's *t*-test with GraphPad Prism 6 (GraphPad Software, USA). In all cases, *P*<0.05 was considered statistically significant.

## Results

### ZIC2 is highly expressed in lung adenocarcinoma tissues and indicated poor prognosis

**Figure 1:**
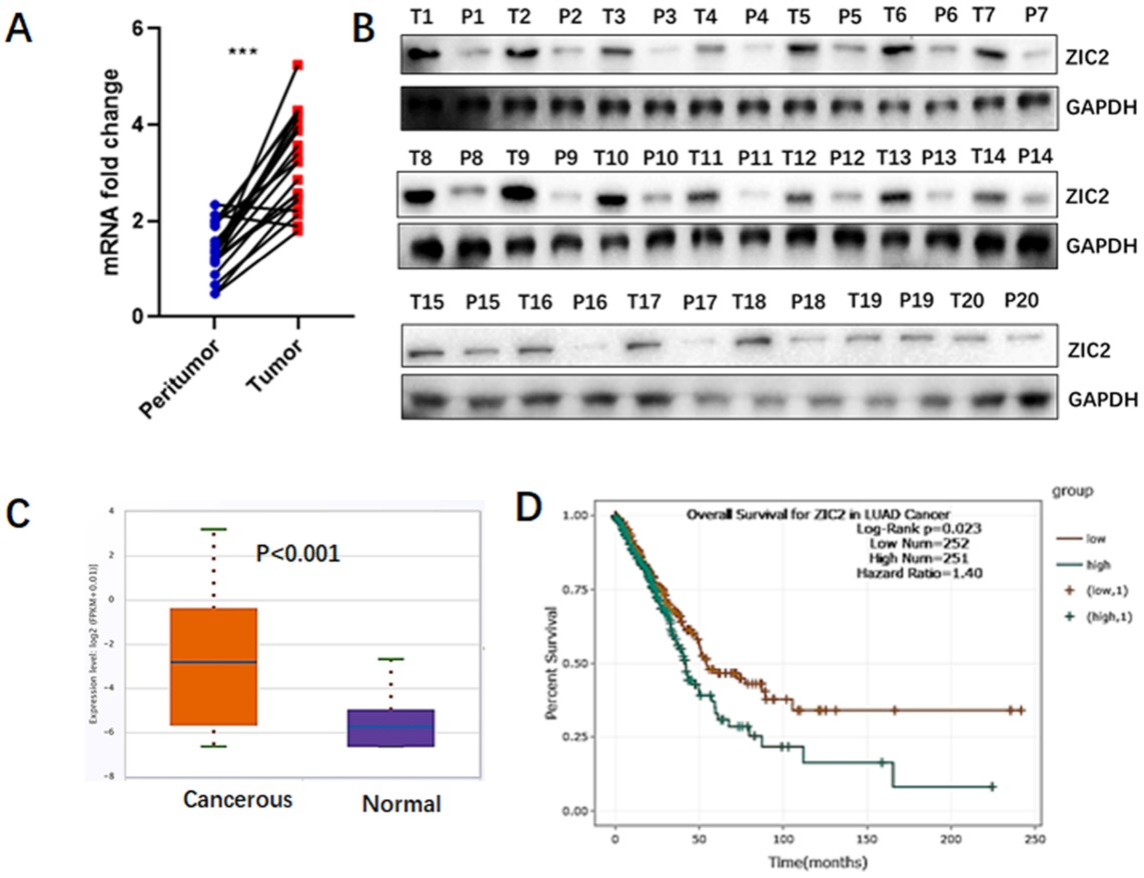
** ZIC2 was preferentially expressed in LAC and indicated poorer prognosis.** (**A**) mRNA expression of ZIC2 in 20 paired LAC and adjacent normal lung tissues was detected by RT-PCR assays. (**B**) Protein expression of ZIC2 in 20 paired LAC and adjacent normal lung tissues was detected by WB assays. (**C**) mRNA expression pattern of ZIC2 between LAC and normal lung tissues were evaluated according to TCGA database. (**D**) Patients with high ZIC2 expression had significantly shorter overall survival according to TCGA database.

To assess the expression of ZIC2 in lung adenocarcinoma, we quantitatively analyzed both mRNA and protein levels of ZIC2 in 20 LAC tissues and the paired non-cancerous lung tissues. As shown in Figure 1A, RT-PCR assays showed that, compared with non-cancerous tissues, mRNA expression of ZIC2 was highly expressed in LAC tissues. Consistently, we found that protein expression of ZIC2 was also upregulated in tumor tissues (Figure 1B). Furthermore, according to TCGA database, ZIC2 mRNA expression was significantly increased in lung adenocarcinoma tumor tissues compared with normal lung tissues (*P*<0.001) (Figure 1C). In addition, the K-M plot analysis showed that LAC patients with high ZIC2 expression had a significantly shorter overall survival (*P*=0.023) (Figure 1D). Collectively, above data indicated ZIC2 was overexpressed in LAC tissues and closely associated with poorer prognosis.

### Knockdown of ZIC2 suppresses the stem cell-like phenotype in LAC

**Figure 2:**
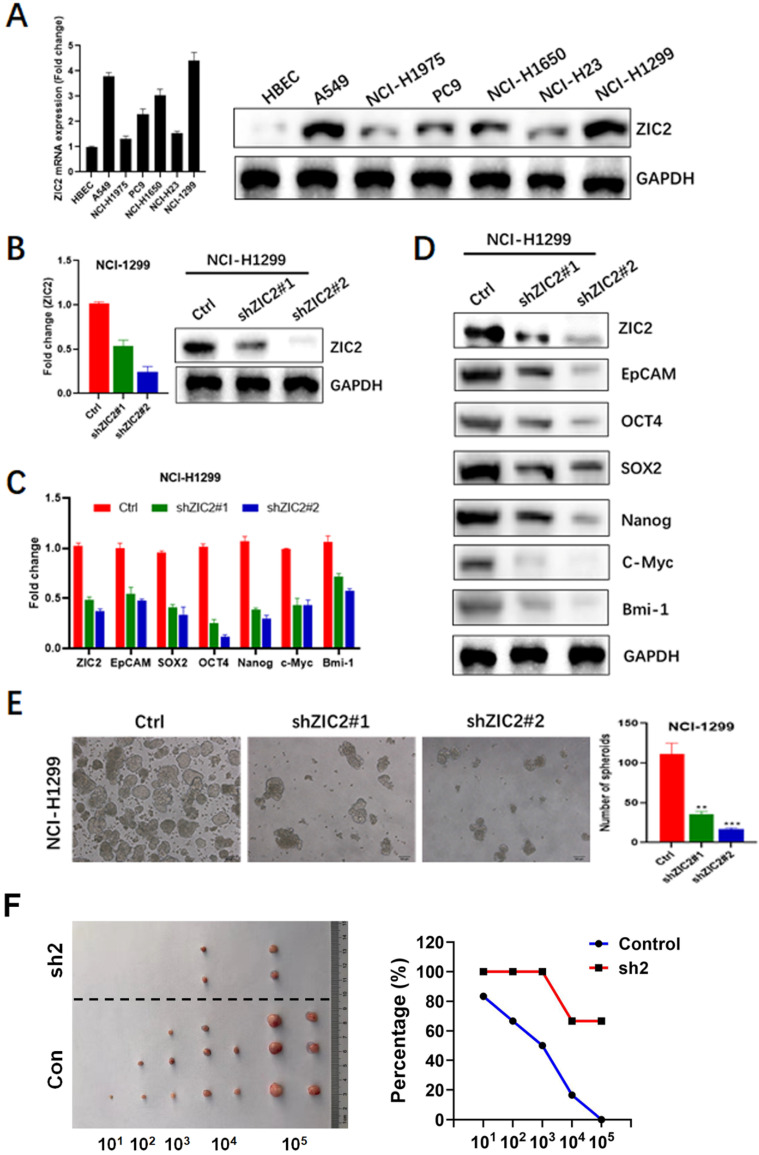
** ZIC2 knockdown restricted CSC-associated phenotype in LAC cells.** (**A**) Expression levels of ZIC2 in normal and LAC cell lines were determined by RT-PCR and WB assays. (**B**) ZIC2 knockdown efficiencies were evaluated by RT-PCR (left) and WB (right) assays. (**C**) Effects of ZIC2 knockdown on CSC-associated marker expression in NCI-H1299 cells were evaluated by RT-PCR assays. (**D**) WB results for describing the effects of ZIC2 knockdown on CSC-associated marker expression in NCI-H1299 cells. (**E**) Effects of ZIC2 knockdown on sphere-forming capacity in NCI-H1299 cells; Representative images were shown in left panel. (**F**) Ratio of tumor-free mice after 12 weeks' tumor formation after injection of indicated numbers of LAC cells. Images were shown in the left panel.

To further investigate the role of ZIC2 in LAC, we firstly detected the expression of ZIC2 in several LAC cell lines including A549, NCI-H1975, PC9, NCI-H1650, NCI-H23, and NCI-H1299, as well as a normal bronchial epithelial cell line, HBEC. As shown in Figure 2A, ZIC2 showed was almost undetected in HBEC, whereas showed detectable levels in all LAC cell lines. Among all LAC cell lines we used, NCI-H1299 cells exerted the highest expression level of ZIC2, while NCI-H1975 cell line showed the lowest expression. Therefore, ZIC2 was knockdown in NCI-H1299 cells while NCI-H1975 was selected for further ZIC2 overexpression.

Two distinct shRNAs targeting ZIC2 were transfected into NCI-H1299 cells respectively, and RT-PCR as well as WB assays were conducted to evaluate the knockdown efficiencies. Results showed shRNA2 achieved a more powerful knockdown efficiency, whereas both two shRNAs achieved satisfactory efficiencies (>50%) (Figure 2B). Therefore, we first observed the effects of ZIC2 on expressions of CSC-associated markers including EpCAM, OCT4, SOX2, NANOG, C-Myc and Bmi-1. Results showed that ZIC2 knockdown significantly reduced mRNA expression of these CSC-associated marker expression in NCI-H1299 cells (Figure 2C). Consistently, WB assays confirmed the findings of RT-PCR assays (Figure 2D). Next, the effect of ZIC2 expression on sphere-forming capacity of LAC cells was assessed. We found that downregulation of ZIC2 greatly decreased the numbers of spheroid cells (Figure 2E). Last, we examined effects of ZIC2 on CSC traits via serial tumorigenesis experiments. Results showed that ZIC2 knockdown obviously inhibited tumor formation at all densities we tested (Figure 2F), confirming the critical role of ZIC2 in maintaining CSCs traits in NCI-H1299 cells.

### Knockdown of ZIC2 sensitized LAC cells to conventional chemotherapy regimens

**Figure 3:**
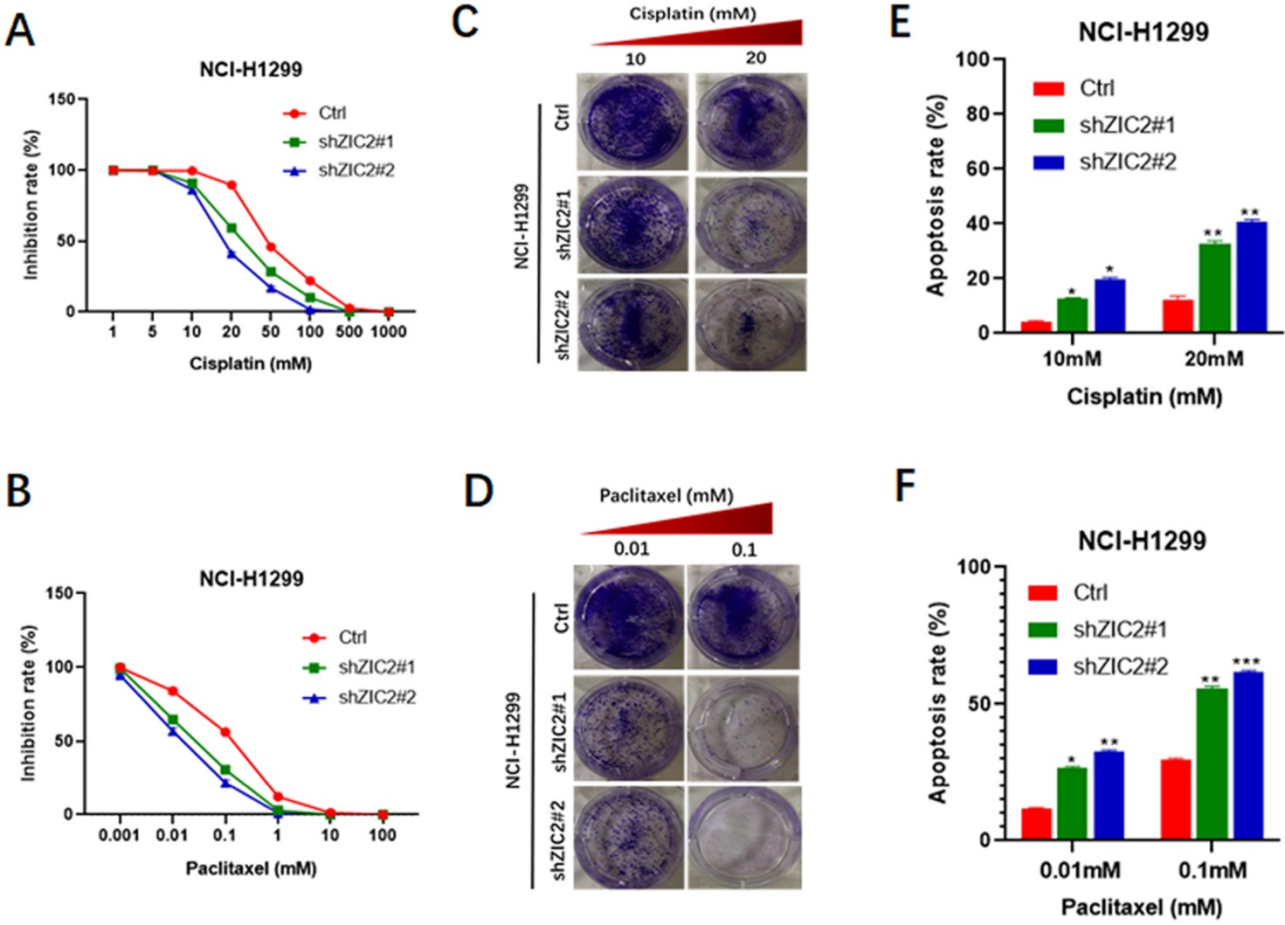
** ZIC2 knockdown sensitized NCI-H1299 cells to cisplatin and paclitaxel treatment.** (**A**) Effects of ZIC2 knockdown on the response to different concentrations of cisplatin in NCI-H1299 cells were evaluated by CCK-8 assays. (**B**) Effects of ZIC2 knockdown on the response to different concentrations of paclitaxel in NCI-H1299 cells were evaluated by CCK-8 assays. (**C**) Colony formation assays were conducted to evaluate the effects of ZIC knockdown on cisplatin resistance in NCI-H1299 cells; two concentrations (10 and 20 mM) were selected according to the results of CCK-8 assays. (**D**) Colony formation assays were conducted to evaluate the effects of ZIC knockdown on paclitaxel resistance in NCI-H1299 cells; two concentrations (0.01 and 1 mM) were selected according to the results of CCK-8 assays. (**E**) Apoptosis rates of ZIC2-KD and corresponding parental NCI-H1299 cells under different concentrations of cisplatin treatments were determined by FACS. (**F**) Apoptosis rates of ZIC2-KD and corresponding parental NCI-H1299 cells under different concentrations of paclitaxel treatments were determined by FACS.

It is well acknowledged that chemoresistance acts as a hallmark of stem cell-like trait 22, thus, we also investigated the involvement of ZIC2 in drug resistance of lung adenocarcinoma cells. CCK8 assays were performed to evaluate the inhibitory rates of LAC cells under different drug concentration treatment. We found that ZIC2 knockdown resulted in a more significant reduced inhibition rates when treated with cisplatin in a wide range of concentrations (from 5μM to 500μm), compared with the control cells (Figure 3A). Consistently, ZIC2 knockdown cells also sensitized NCI-H1299 cells to paclitaxel treatment (Figure 3B). To further verify above findings, we chose the two significant inhibitory concentration of cisplatin (10um, 20um) and paclitaxel (0.01um and 0.1um) to treat LAC cells, and cell proliferation potentials under chemotherapy regimen treatment were assessed by colony formation assays. We found that ZIC2 knockdown sensitized LAC cells to either cisplatin or paclitaxel treatment at both two concentrations, evidenced by decreased number of clones formed (Figure 3C and D). Finally, to consolidate our previous results, flow cytometry assays were performed. Results showed that, compared with control cells, ZIC2 knockdown resulted in significantly higher apoptosis rates of LAC cells when treated with certain concentration of cisplatin or paclitaxel (Figure 3E and F). Therefore, our data demonstrate that ZIC knockdown significantly sensitized LAC cells to conventional chemotherapy regiment treatments.

### Overexpression of ZIC2 promotes stem cell-like phenotype in LAC

**Figure 4:**
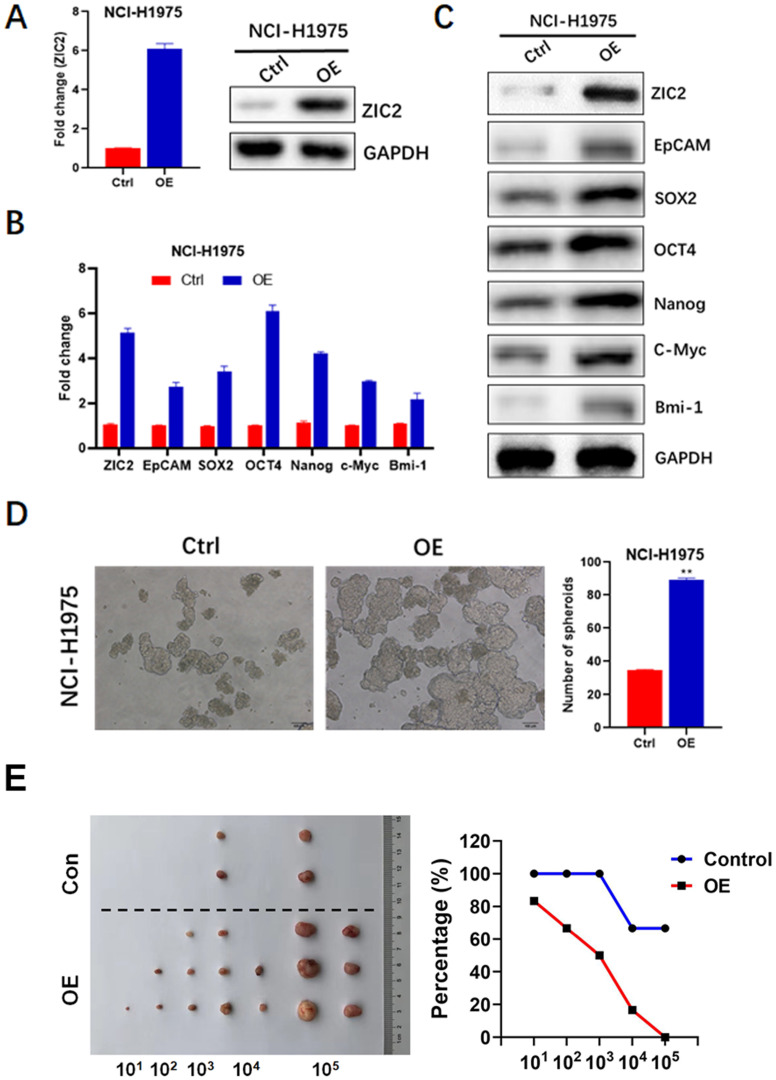
** ZIC2 overexpression promoted CSC-associated phenotype in NCI-H1975 cells.** (**A**) Efficiencies of ZIC2 overexpression in NCI-H1975 cells were validated by RT-PCR and WB assays. (**B**) Effects of ZIC2 overexpression on CSC-associated marker expression were determined by RT-PCR assays. (**C**) Effects of ZIC2 overexpression on CSC-associated marker expression were determined by WB assays. (**D**) Effects of ZIC2 overexpression on sphere-forming capacity in NCI-H1975 cells were evaluated; Representative images were shown as left panel. (**F**) Ratio of tumor-free mice after 12 weeks' tumor formation after injection of indicated numbers of LAC cells. Images were shown in the left panel.

To confirm the role of ZIC2 in LAC CSC maintenance and expansion, we successfully constructed the lentiviral-based stable ZIC2 overexpressed NCI-H1975 cells, confirmed by significantly increased ZIC2 expression according to RT-PCR and WB assays (Figure 4A). Afterwards, expressions of CSC-associated markers, sphere-forming, and drug resistance capacities were further investigated. ZIC2 overexpression resulted in increased expression levels of EpCAM, OCT4, SOX2, NANOG, C-Myc and Bmi-1 according to results of RT-PCR and WB assays (Figure 4B and C). Of note, the ZIC2-OE NCI-H1975 cells showed an increased sphere-forming numbers when compared with control cells (Figure 4D). Thus, our data indicated that forced expression of ZIC2 in LAC cells drove these cells towards a CSC-like state. Finally, results from serial tumorigenesis experiments showed that ZIC2 overexpression greatly enhanced tumor formation capacity of NCI-H1975 cells at all densities we tested (Figure 2F).

### Overexpression of ZIC2 renders LAC cells with resistance to conventional chemotherapy regimens

**Figure 5:**
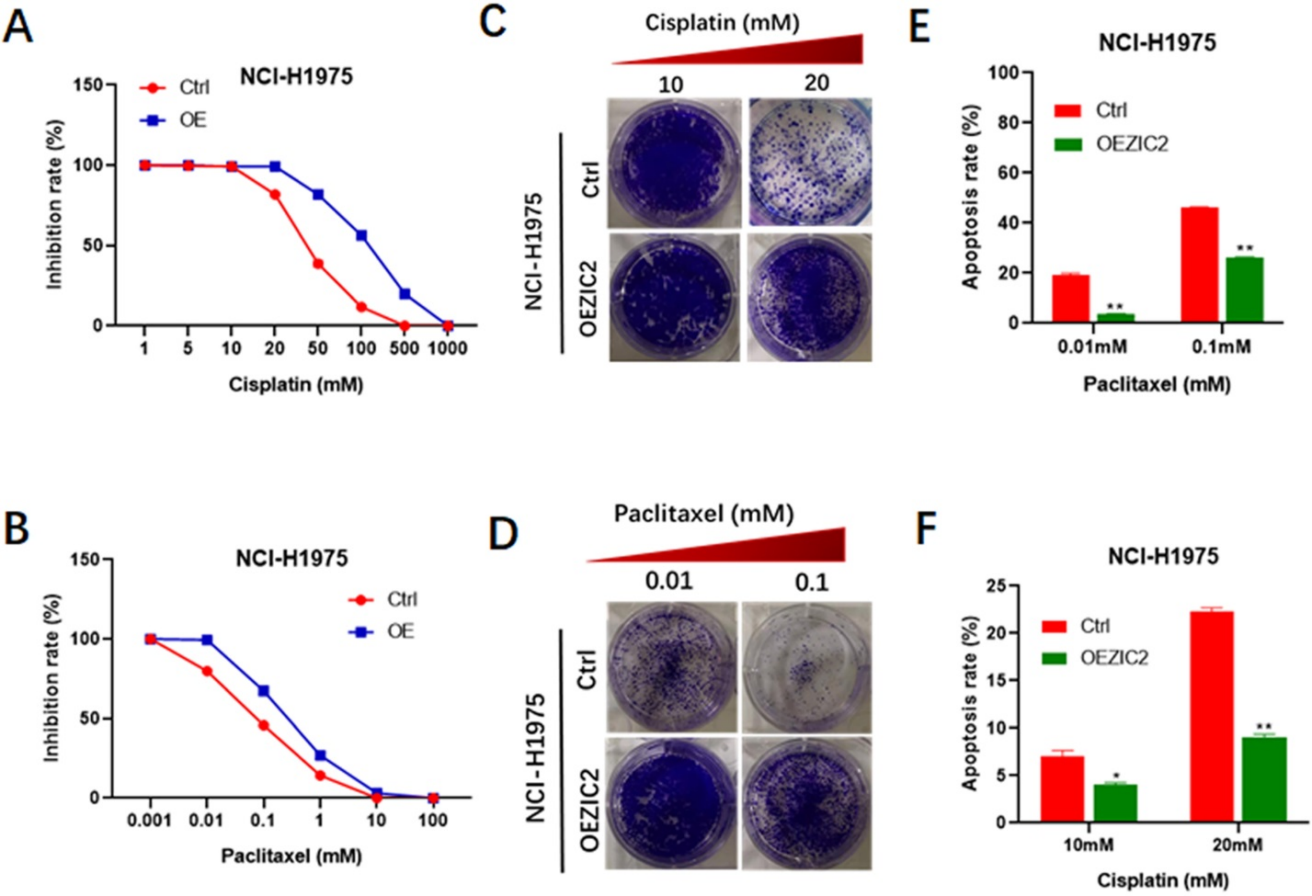
** ZIC2 overexpression induced resistance to cisplatin and paclitaxel in NCI-H1975 cells.** (**A**) Effects of ZIC2 overexpression on the response to different concentrations of cisplatin in NCI-H1975 cells were evaluated by CCK-8 assays. (**B**) Effects of ZIC2 overexpression on the response to different concentrations of paclitaxel in NCI-H1975 cells were evaluated by CCK-8 assays. (**C**) Colony formation assays were conducted to evaluate the effects of ZIC overexpression on cisplatin resistance in NCI-H1975 cells; two concentrations (10 and 20 mM) were selected according to the results of CCK-8 assays. (**D**) Colony formation assays were conducted to evaluate the effects of ZIC overexpression on paclitaxel resistance in NCI-H1975 cells; two concentrations (10 and 20 mM) were selected according to the results of CCK-8 assays. (**E**) Apoptosis rates of ZIC2-OE and corresponding parental NCI-H1299 cells under different concentrations of cisplatin treatments were determined by FACS. (**F**) Apoptosis rates of ZIC2-OE and corresponding parental NCI-H1299 cells under different concentrations of paclitaxel treatments were determined by FACS.

Next, we set out to investigate the impacts of force expression of ZIC2 on drug resistance. CCK8 assays indicated that ZIC2-overexpressed cells exhibited a more significant higher resistance to various concentrations of cisplatin treatment (from 5μM to 500μm), compared with control cells (Figure 5A). Similarly, ZIC2 overexpression also sensitized NCI-H1299 cells to different concentrations of paclitaxel treatment (Figure 5B). Colony-formation assays indicated that ZIC2 overexpression conferred considerable cisplatin and paclitaxel resistance capacities to LAC cells, evidenced by increased number of clones formed under same regimen treatment (Figure 5C and D). Finally, FACS assays indicated that compared with control cells, ZIC2 overexpression resulted in significantly lower apoptosis rates of LAC cells when treated with certain concentration of cisplatin or paclitaxel (Figure 5E and F). Taken together, these data demonstrate that forced ZIC2 expression significantly sensitized LAC cells to conventional chemotherapy regiment treatments.

### ZIC2 enhances CSC traits via an OCT4-dependent manner

**Figure 6:**
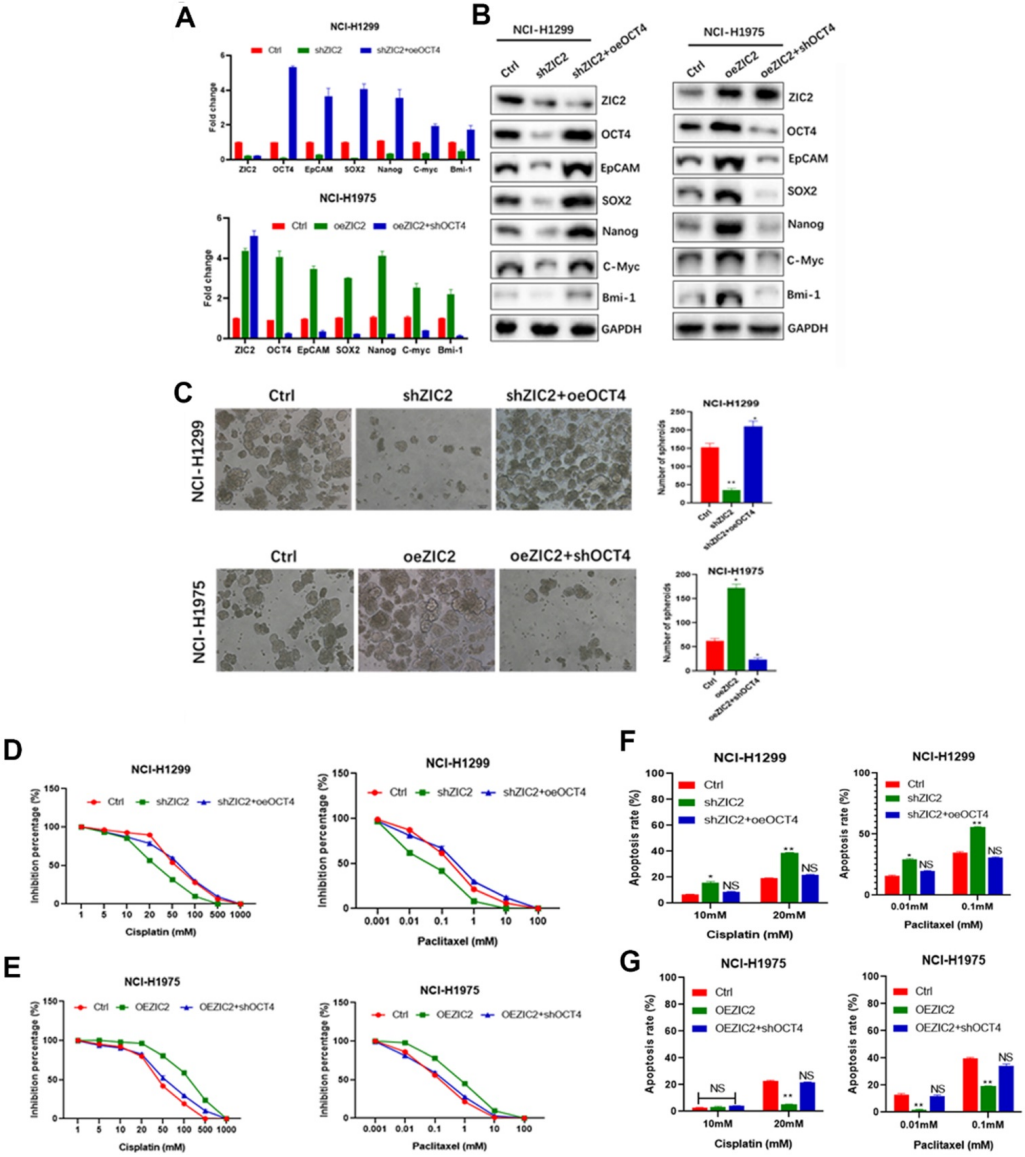
** ZIC2 promoted CSC traits in LAC vie up-regulating OCT4 expression.** (**A**) OCT4 expression was restored in ZIC2-KD NCI-H1299 cells (upper), or silenced in ZIC2-OE NCI-H1975 cells (lower), and their impacts on CSC-associated marker expression were evaluated by RT-CPR. (**B**) OCT4 expression was restored in ZIC2-KD NCI-H1299 cells (left), or silenced in ZIC2-OE NCI-H1975 cells (right), and their impacts on CSC-associated marker expression were evaluated by WB assays. (**C**) OCT4 expression was restored in ZIC2-KD NCI-H1299 cells (upper), or silenced in ZIC2-OE NCI-H1975 cells (lower), and their impacts on sphere-forming capacities were evaluated. (**D**) OCT4 expression was restored in ZIC2-KD NCI-H1299 cells, and its impacts on the response to different concentrations of cisplatin (left) or paclitaxel (right) were detected by CCK-8 assays. (**E**) OCT4 expression was silenced in ZIC2-OE NCI-H1975 cells, and its impacts on the response to different concentrations of cisplatin (left) or paclitaxel (right) were detected by CCK-8 assays. (**F**) OCT4 expression was restored in ZIC2-KD NCI-H1299 cells, and its impact on apoptosis rate under different concentrations of cisplatin (left) or paclitaxel (right) treatment was determined by FACS. (**G**) OCT4 expression was silenced in ZIC2-OE NCI-H1975 cells, and its impact on apoptosis rate under different concentrations of cisplatin (left) or paclitaxel (right) treatment was determined by FACS.

Among the numerous stemness-related molecules associated with ZIC2 expression, we focused on OCT4, which showed the most significant mRNA expression changes due to ZIC2 alterations. Also, OCT4 was reported to play pivotal role for CSC properties in LAC 17. To investigate interaction of ZIC2 with OCT4 in LAC CSCs, we overexpressed OCT4 in ZIC2-KD NCI-H1299 cells, while silenced OCT4 expression in ZIC2-OE NCI-H1975 cells. As expected, forced expression of OCT4 restored expression of CSC-associated markers in ZIC2-KD NCI-H1299 cells, whereas knocking down OCT4 greatly abolished the promotional effects of ZIC2 overexpression on CSC-associated marker expression (Figure 6A and B). Moreover, OCT4 overexpression successfully rescued the sphere-forming capacity in NCI-H1299 cells, evidenced by increased number of spheroids formed. On the contrary, OCT4 silence dramatically attenuated the sphere-forming capacity rendered by ZIC2 overexpression in NCI-H1975 cells (Figure 6C).

More importantly, upregulation of OCT4 reversed the ZIC2 knockdown-induced cisplatin and paclitaxel sensitization in NCI-H1299 cells according to CCK-8 assays (Figure 6D), whereas downregulation of OCT4 significantly abolished ZIC2-mediated cisplatin and paclitaxel resistance in NCI-H1975 cells (Figure 6E). In concordance with the results of CCK-8 assay, FACS results demonstrated that the apoptosis rates of ZIC2-KD NCI-H1299 cells under cisplatin or paclitaxel treatments were significantly reduced upon OCT4 overexpression (Figure 6F). Contrarily, OCT4 knockdown increased the apoptosis rates in ZIC2-OE NCI-H1975 cells at both two concentrations (Figure 6G). Together, our findings strongly indicated that ZIC2 promoted CSC traits in LAC via up-regulating OCT4 expression.

## Discussion

ZIC2 is a gene originally identified by their homology to the drosophila odd-paired genes. Heterozygous deletions and mutations of ZIC2 can cause severe brain malformation 23, 24. Thus, it is critical for the development of CNS 25. Recent reports have shown a correlation between ZIC2 expression and carcinogenesis. ZIC2 is aberrantly activated in various cancer types, such as hepatocellular carcinoma 19, bladder cancer 26, and cervical cancer 27. ZIC2 is upregulated in these tumors and is associated with a higher stage of tumors and poor prognosis. Therefore, ZIC2 is regarded as an oncogene. However, there are no data regarding the expression of ZIC2 in LAC and its clinical significance. In our study, we revealed that ZIC2 was highly expressed in LAC tumor tissues and indicated a poor overall survival of LAC patients, which suggested that ZIC2 may be a potential prognostic biomarker for LAC patients.

CSCs have been identified in many solid tumors and play a central role in tumor initiation, progression, and therapeutic resistance 28. A better understanding of the molecular mechanisms essential for CSC maintenance and expansion would shed light on the enhancement of clinical management. Importantly, specifically and effectively targeting CSCs is widely considered as a potential strategy to improve the overall survival rates of cancer patients 19, 29. Previous studies revealed that ZIC2 was required for the self-renewal maintenance of liver CSCs 19. Additionally, a recent report showed that ZIC2 is highly expressed in cervical cancer and associated with activation of Hedgehog signaling, a crucial cancer stemness pathway 27. However, the LAC CSC biology remains largely unknown and whether ZIC2 play a role in LAC CSC has not been reported previously. Here, using both loss-of-function and gain-of-function experiments, we provide evidence for the role of ZIC2 in the regulation of LAC cell self-renewal and stemness. We found ZIC2 expression was positively associated with the expression of stemmed-related molecules and was essential for the spheroid cell formation.

The development of chemoresistance is a major obstacle for the effective treatment of human malignancies 30. Cisplatin and paclitaxel are fist-line chemotherapeutic agents used for various cancers 31. However, the response to these drugs for LAC patients soon disappears, leading to patient death and the poor five-year survival rates 32. Chemoresistance is a complex phenomenon and revealing the molecular mechanisms related to inherent and acquired resistance is important to overcome drug resistance. Considering that cancer stem cells are responsible for the tumor chemoresistance and the functional roles of ZIC2 in CSCs, the influence of ZIC2 on cisplatin and paclitaxel resistance needed to be deeply investigated in lung adenocarcinoma cells. Our study found that ZIC2 overexpression cells dramatically enhanced drug resistance properties in LAC cells. Therefore, our study identified a novel therapeutic target for reversing drug resistance in LAC, and developing strategy to lower ZIC2 expression may increase the efficacy of cisplatin and paclitaxel treatment in LAC tumor.

OCT4 was identified as a stem cell transcription factor and had been reported to be highly expressed in LAC tumor 12, 17. OCT4 acts as a gatekeeper in the beginning of mammalian development and regulates the transcription of many genes, such as SOX2, NANOG and c-MYC 12. Indeed, OCT4 was the key gene between tumor initiation and progression via the direct mediation of key molecules that regulate cancer stemness traits 33. Moreover, high OCT4 expression was found to be closely associated with poor outcomes in LAC patients 17. ZIC2, as transcription factor (TF), behaved as the other core pluripotent TFs (SOX2, NANOG, c-MYC) in cancer stem cells 24. Interestingly, the association between ZIC2 and OCT4 is reported in liver cancer 19. In addition, ZIC2 is required for the expression of OCT4. Not surprisingly, lung cancer subtypes may also share core regulatory genes and common signaling pathways. Our *in vitro* assays further demonstrated that ZIC2 acted as a critical oncogene responsible for self-renewal and stemness of LAC by upregulation of OCT4. Therefore, ZIC2-induced OCT4 may be considered as a master regulator to maintain and govern LAC CSCs. This suggests that the regulatory pathway between ZIC2 and OCT4 may be significant for the stabilization of stemness-like state.

In summary, our study provided various evidence to link high expression of ZIC2 with the acquisition of LAC CSC-like properties, and proposed a novel mechanism of LAC CSC-like traits mediated by ZIC2. We unveiled that ZIC2 maintained the self-renewal, stemness and chemoresistance capacities in an OCT4-dependent manner. To our knowledge, this is the first study to reveal that ZIC2 exerted dramatic effects on CSC-like properties in human LAC tumor. The ZIC2-OCT4 network will facilitate the evaluation of the potential therapeutic efficacy of chemotherapy and predict patient sensitivity to treatment.

## Supplementary Material

Supplementary figures and tables.
