# Supplementary material for: ZIC2 promotes cancer stem cell traits via up-regulating OCT4 expression in lung adenocarcinoma cells
Source: J Cancer. 2020 Aug 19;11(20):6070–80. doi: 10.7150/jca.44367 (PMC7477430; doi:10.7150/jca.44367)
Supplement: Supplementary file 1 — Supplementary figures and tables. [file jcav11p6070s1.pdf]

Supplementary Figure 1

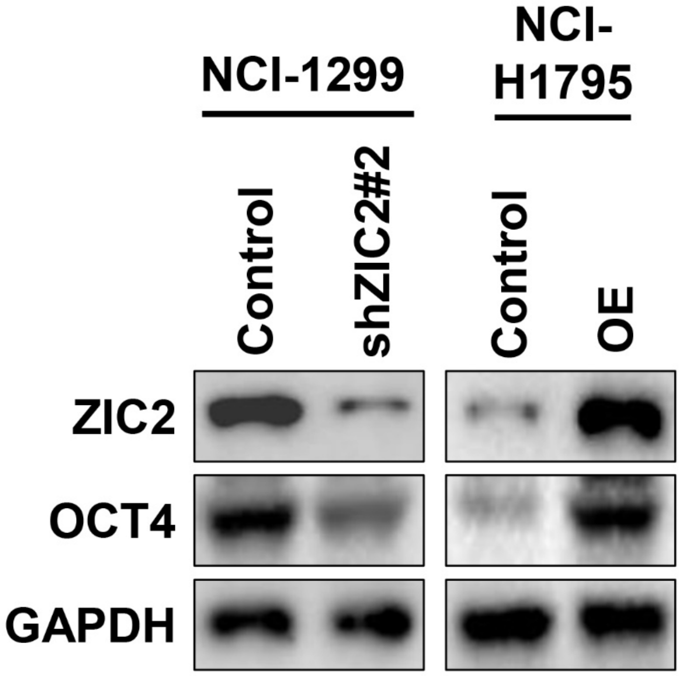

Supplementary Figure 1. Validations of ZIC2 expression manipulations before in vivo experiments via WB assays.

Supplementary Figure 2

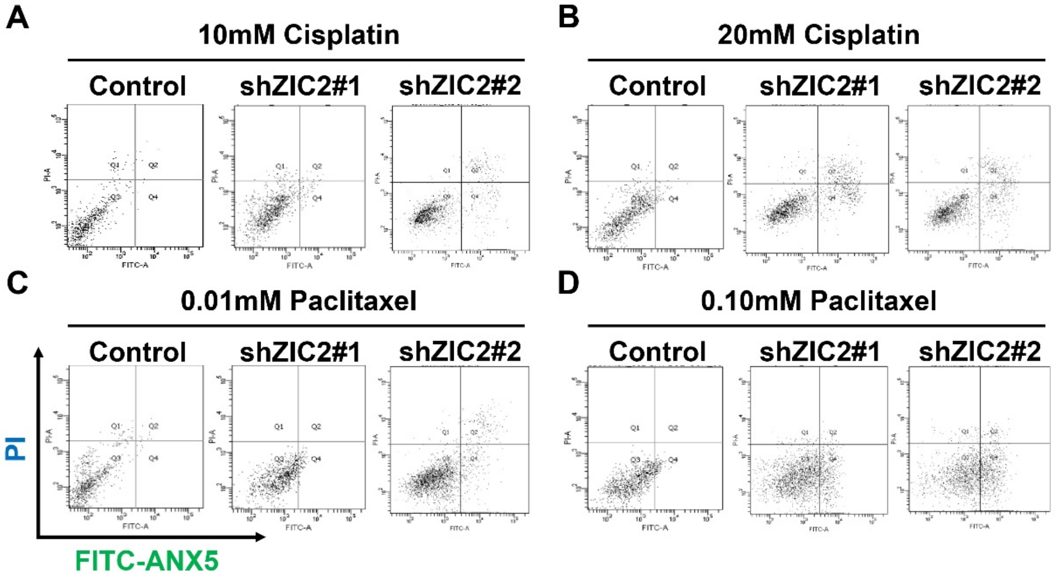

Supplementary Figure 2. Representative images of apoptosis assays after indicated regimen treatment, which is corresponded to Figure 3. (A) Representative images of apoptosis assays after 10mM Cisplatin treatment. (B) Representative images of apoptosis assays after 20mM Cisplatin treatment. (C) Representative images of apoptosis assays after 0.01mM Paclitaxel treatment. (D) Representative images of apoptosis assays after 0.10mM Paclitaxel treatment.

Supplementary Figure 3

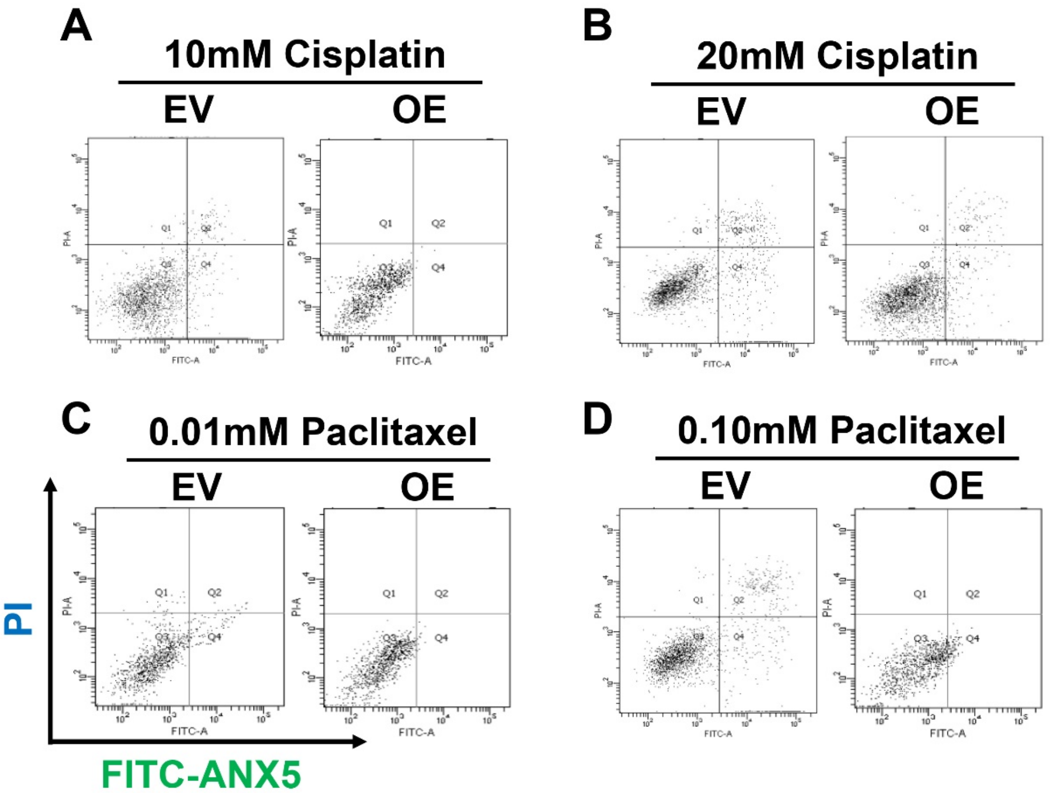

Supplementary Figure 3. Representative images of apoptosis assays after indicated regimen treatment, which was corresponded to Figure 5. (A) Representative images of apoptosis assays after 10mM Cisplatin treatment. (B) Representative images of apoptosis assays after 20mM Cisplatin treatment. (C) Representative images of apoptosis assays after 0.01mM Paclitaxel treatment. (D) Representative images of apoptosis assays after 0.10mM Paclitaxel treatment.

Supplementary Figure 4

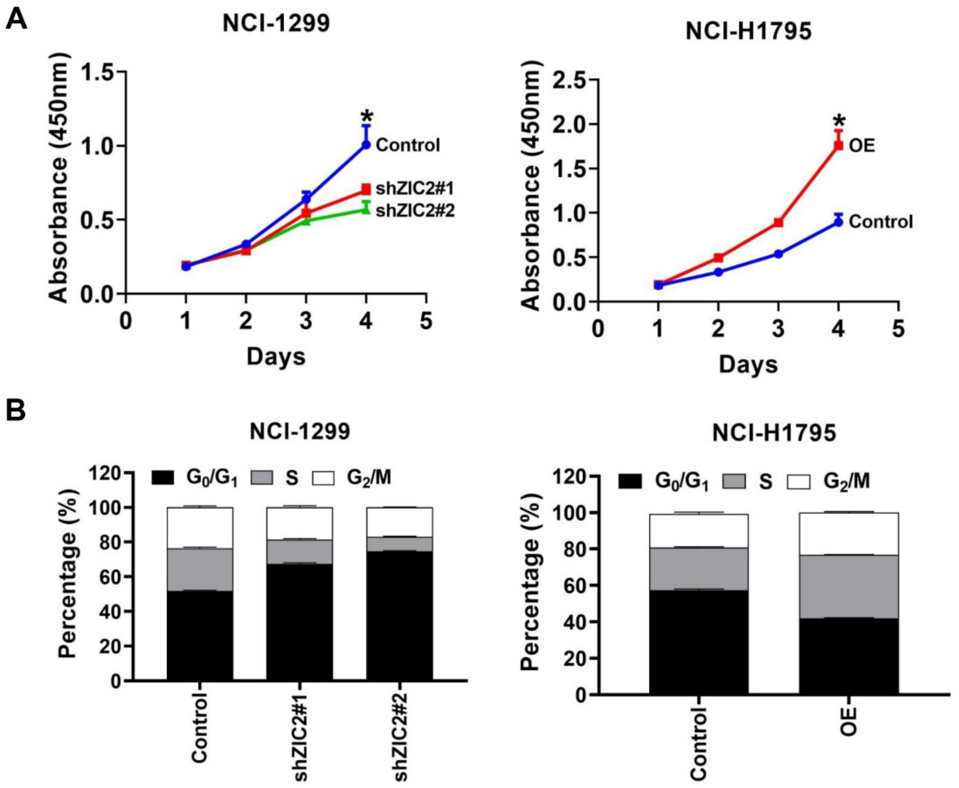

Supplementary Figure 4. Effects of ZIC2 on LAC proliferation and cell cycle. (A) Effects of ZIC2 expression on LAC proliferation. (B) Effects of ZIC2 expression on cell cycle of LAC cells.

Supplementary Figure 5

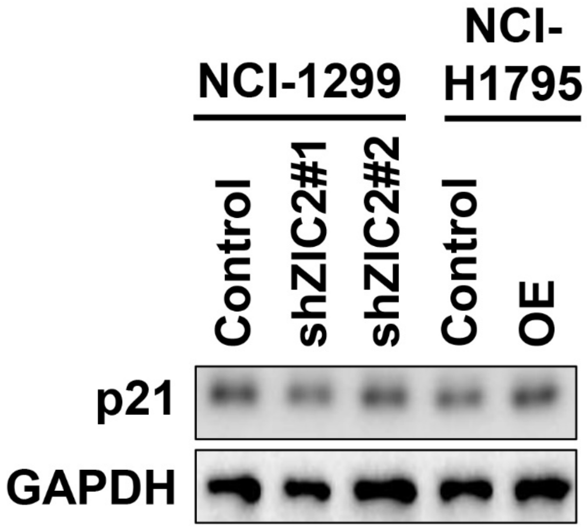

Supplementary Figure 5. ZIC2 shed no significant influence on p21 protein expression.

Table S1. Primers used in present study

Gene name	Forward 5'-3'	Reverse 5'-3'
ZIC2	GCGCAACTCCACAACCAGTA	TGCCGCATATAGCGGAAAAAG
EpCAM	AATCGTCAATGCCAGTGTACTT	TCTCATCGCAGTCAGGATCATAA
Sox2	CTCGTGCAGTTCTACTCGTCG	AGCTCTCGGTCAGGTCCTTT
OCT4	GTGTTCAGCCAAAAGACCATCT	GGCCTGCATGAGGGTTTCT
Nanog	TCCCGAGAAAAGATTAGTCAGCA	AGTGGGGCACCTGTTTAACTT
c-Myc	ATGGCCCATTACAAAGCCG	TTTCTGGAGTAGCAGCTCCTAA
Bmi-1	CGTGTATTGTTCGTTACCTGGA	TTCAGTAGTGGTCTGGTCTTGT
GAPDH	TGTGGGCATCAATGGATTTGG	ACACCATGTATTCCGGGTCAAT

Table S2. Information of antibodies used in present study

Target protein	Manufacturer	Application	Dilution
ZIC2	Abcam (ab150404)	WB	1:1000
EpCAM	Abcam (ab223582)	WB	1:1000
Oct4	Abcam (ab19857)	WB	1:1000
SOX2	Abcam (ab93689)	WB	1:1000
Nanog	Abcam (ab109250)	WB	1:1000
c-Myc	Abcam (ab168727)	WB	1:1000
Bmi-1	Abcam (ab38295)	WB	1:1000
GAPDH	CST (#5174)	WB	1:2000
